# The landscape of variants in pre-mRNA-processing factor genes in an Irish cohort

**DOI:** 10.1016/j.gimo.2026.104402

**Published:** 2026-05-06

**Authors:** Laura K. Finnegan, Anna R. Ridgeway, Matthew Carrigan, Hilary Dempsey, Róisín Long, Evan Matthews, Daan Panneman, Jacqueline Turner, Adrian Dockery, Laura Whelan, Ciara Shortall, Ella Kopčić, Frans P.M. Cremers, Susanne Roosing, Claire Kirk, Giuliana Silvestri, David Keegan, Paul F. Kenna, Emma Duignan, Naomi Chadderton, G. Jane Farrar

**Affiliations:** 1 School of Genetics and Microbiology, Trinity College Dublin, Dublin 2, Ireland; 2 The Research Foundation, Royal Victoria Eye and Ear Hospital, Dublin 2, Ireland; 3 Department of Human Genetics, Radboud University Medical Center, Nijmegen, The Netherlands; 4 Mater Clinical Ophthalmic Genetics Unit, The Mater Misericordiae University Hospital, Dublin 7, Ireland; 5 Department of Ophthalmology, The Royal Victoria Hospital, Belfast, United Kingdom; 6 Department of Ophthalmology, Royal Victoria Eye and Ear Hospital, Dublin 2, Ireland

**Keywords:** Autosomal dominant retinitis pigmentosa (adRP), mRNA-processing, Variant classification

## Abstract

**Purpose:**

Variants in pre-messenger RNA (mRNA) processing genes account for ∼15% to 20% of autosomal dominant retinitis pigmentosa, causing severe vision loss. We provide an in-depth characterization of pre-mRNA-processing variants in the population, with a focus on genotype-phenotype correlations.

**Methods:**

Participants underwent next-generation sequencing, variant filtering and interpretation. Impact(s) on protein structure/function were predicted through AlphaFold, ColabFold, among others. Clinical information was collated and segregation analysis undertaken. Variants were classified according to American College of Medical Genetics and Genomics/Association for Molecular Pathology guidelines.

**Results:**

A total of 635 patients with retinitis pigmentosa (RP) were screened. Notably, 36 different variants were identified in *PRPF31*, *PRPF8*, *SNRNP200*, *PRPF3*, *RP9*, and *PRPF6*; 19 were classified as likely pathogenic/pathogenic. Thirteen of 36 variants were absent from PubMed, gnomAD, LOVD, and ClinVar, with an additional 10 not previously associated with retinal degeneration. Genotype-phenotypes correlations were illuminated; variants in PRPF genes caused more severe retinal degeneration than *SNRNP200* variants.

**Conclusion:**

We provide a detailed overview of the genetic landscape of variants in pre-mRNA-processing factor genes in Ireland, accounting for 14% of genetically solved autosomal dominant retinitis pigmentosa cases. Results highlight significant genetic diversity between populations, mutational diversity inherent in inherited retinal degenerations and the role of variants in mRNA-processing genes as causative of devastating ocular disorders.

## Graphical abstract

**Figure:**
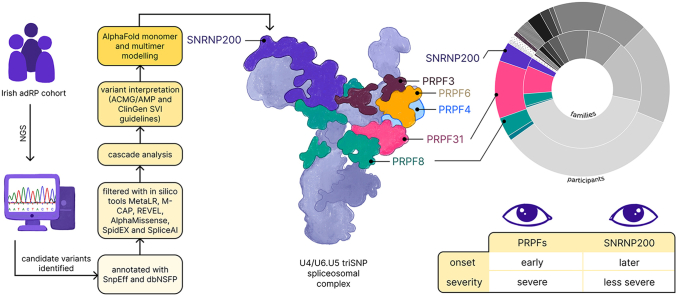

## Introduction

Retinitis pigmentosa (RP) is the most common inherited retinal degeneration (IRD) which initially manifests with reduced night vision and visual field loss, progressing to central and color-vision deficits due to secondary cone degeneration, frequently leading to legal blindness. IRDs are highly genetically heterogeneous, with causative variants in 468 genes reported to date, >100 of which are associated with RP and of these, 15 genes are currently known to be causative of adRP (RetNet, https://sph.uth.edu/RetNet/, accessed 14 July 2025).^1^^,^^2^ Of note, typically 15% to 20% of adRP is attributable to variants in pre-messenger RNA (mRNA) processing genes.^3^ Here, we describe the landscape of candidate disease-causing variants in these genes in an Irish cohort of individuals with IRDs.

Typically, mammalian genes comprise exons, introns and untranslated regions. Splicing, involving removal of introns to form mature RNA transcripts, is orchestrated by the spliceosome. The spliceosome core is composed of 5 small nuclear RNAs (snRNAs)-U1, U2, U4, U5, and U6, each of which is associated with small nuclear proteins (snRNPs) and non-snRNP proteins.^4^ U1 and U2 complexes recognize 5ʹ and 3ʹ intronic splice sites, whereas U4/U6.U5 tri-snRNPs form the precatalytic spliceosomal complex.^5^ Several genes encoding spliceosome-associated proteins have been associated with RP: *PRPF3* (HGNC:17348), *PRPF4* (HGNC:17349), *PRPF6* (HGNC:15860), *PRPF8* (HGNC:17340), *PRPF31* (HGNC:15446), *PRPF38A* (HGNC:25930), *SNRNP200* (HGNC:30859), *RP9 (PAP1)* (HGNC:10288), *CWC27* (HGNC:10664), *USH1G* (HGNC:16356), *DHX38 (PRPF16)* (HGNC:17211), *RNU4-2* (HGNC:10193), *RNU6-1* (HGNC:10227), *RNU6-2* (HGNC:34270), *RNU6-8* (HGNC:34285), and *RNU6-9* (HGNC:34269).^6^^,^^7^ Pre-mRNA-processing factors (PRPFs) form part of the U4/U6.U5 tri-snRNP complex, with pathogenic variants in these genes resulting in perturbed stoichiometry of snRNAs and altered composition of the tri-snRNP complex. *SNRNP200*’s encoded protein participates in unwinding U4/U6 snRNAs during assembly and disassembly of the spliceosome.^8^ The precise roles of splicing factors *RP9*, *CWC27*, *USH1G*, and *DHX38* are yet to be elucidated.

Interestingly, although PRPFs are ubiquitously expressed, the retina is primarily affected by variants in these genes. The retina expresses up to 7× more major snRNAs and 2× more minor snRNAs compared with many other tissues, suggesting that splicing may be more active in the retina and variants affecting this process may have greater impact.^9^ Another hypothesis is that retinal genes have weaker 3ʹ and 5ʹ splice sites and therefore, may be more severely affected by alternative splicing when the spliceosome is altered.^10^

Disease mechanisms differ between RNA processing genes. For example, *PRPF31* variants may function via dominant negative mechanism(s) or haploinsufficiency due to loss-of-function (LOF).^11^ Evidence points to the latter because of the prevalence of *PRPF31* whole gene deletions; however, incomplete penetrance adds to the challenge of establishing modes-of-action.^12^ Careful consideration when interpreting *PRPF6* and *PRPF4* variants in adRP has been highlighted. Wang et al^13^ recommends interpreting LOF variants in *PRPF6* with caution because haploinsufficiency has yet to be established as a disease mechanism and to classify missense variants in *PRPF4* and *PRPF31* with similar caution. Elucidating mechanisms of disease is crucial to provide accurate genetic diagnoses and develop safe and effective therapeutics.

Here, we describe the genetic landscape of variants in genes encoding pre-mRNA-processing factors in an Irish cohort of 45 individuals with RP. A total of 36 variants were identified in *PRPF31*, *PRPF8*, *SNRNP200*, *PRPF3*, *RP9*, and *PRPF6* in 34 pedigrees. Of these, 19 are considered to be candidate disease-causing variants identified in *PRPF31*, *PRPF8*, *SNRNP200*, and *PRPF3*. Furthermore, genotype-phenotype correlations are explored, as is the pathogenicity of previously unreported variants. The array of variants identified highlights the significant diversity of variants in RNA processing genes globally that can be causative of IRDs. Additionally, this study provides an updated overview of the genetic landscape of autosomal dominant RP in the Irish population.

## Materials and Methods

### Participant identification and recruitment

Participants were assessed at The Royal Victoria Eye and Ear Hospital, Dublin, The Mater Misericordiae University Hospital, Dublin, and The Royal Victoria Hospital, Belfast and are referred to in this article as participants 1 to 45. Electrodiagnostic and psychophysical testing was performed as previously described.^14^ Ethical approval was granted by hospital ethics committees before commencing the study. The study is part of the Target 5000 study focused on elucidation of the genetic causes of IRDs in Ireland.

### DNA isolation and next-generation sequencing

After the written informed consent procedure, blood or saliva samples were collected from probands and family members. Next-generation sequencing was performed on isolated DNA and analyzed as previously described for target-capture panel sequencing,^15^ for smMIPS panel sequencing^16^ and for genome sequencing.^17^ The UCSC GRCh38/Hg38 genome build was used for target-capture panel sequencing (http://genome.ucsc.edu/cgi-bin/hgGateway?db=hg38), whereas smMIPS and genome sequencing were analyzed using the UCSC GRCh37/Hg19 reference genome (http://genome.ucsc.edu/cgi-bin/hgGateway?db=hg19). All variants in Table 1 have been converted to correspond to the GRCH38/Hg38 reference genome and nomenclature was checked using VariantValidator as detailed in Supplemental Table 1.

**Table 1:** ACMG/ACGS classification for potential disease-causing variants identified in the study

A. Potential Disease-Causing Candidate Variants											
Gene	DNA Variant	Protein Variant	GRCh38 HGVS Genomic Coordinate	Population Data	Computational and Predictive Data	Functional Data	Segregation Data	Other Data	Points	Classification	Previously Identified
*PRPF8*	c.5803C>T	p.(Arg1935Cys)	NC_000017.11:g.1655534G>A	PM2_Supporting + PS4	PM5	PP2	N/A	N/A	8	Likely pathogenic	Yes (PMID:34906470)
*PRPF8*	c.6337_6339del	p.(Lys2113del)	NC_000017.11:g.1653575_1653577del	PM2_Supporting + PS4	PM4	N/A	N/A	N/A	7	Likely pathogenic	Yes (PMID: 34906470, 29099798)
*PRPF8*	c.6928A>G	p.(Arg2310Gly)	NC_000017.11:g.1650882T>C	PM2_Supporting + PS4	PP3_Moderate	PM1	N/A	N/A	9	Likely pathogenic	Yes (PMID:11468273)
*PRPF8*	c.6930G>C	p.(Arg2310Ser)	NC_000017.11:g.1650880C>G	PM2_Supporting + PS4	PS1	PM1	PP1_Strong	N/A	15	Pathogenic	Yes (PMID: 11468273)
*PRPF31*	c.239_249del	p.(Val80GlyfsTer6)	NC_000019.10:g.54121860_54121870del	PM2_Supporting	PVS1	N/A	N/A	N/A	9	Likely pathogenic	No
*PRPF31*	c.240_241insT	p.(Met81TyrfsTer9)	NC_000019.10:g.54121861_54121862insT	PM2_Supporting	PVS1	N/A	N/A	N/A	9	Likely pathogenic	Yes (*PMID:**35456422*)
*PRPF31*	c.528-1G>T	p.?	NC_000019.10:g.54123748G>T	PM2_Supporting	PVS1	N/A	N/A	N/A	9	Likely pathogenic	Yes (*PMID:**29099798*)
*PRPF31*	c.757G>A	p.(Gly253Arg)	NC_000019.10:g.54124558G>A	PM2_Supporting	PP3_Strong	PM1_Supporting	N/A	N/A	6	Likely pathogenic	YesPMID: 33749171
*PRPF31*	c.772_773delinsCAACATGCAACATCAT	p.(Thr258GlnfsTer68)	NC_000019.10:g.54124573_54124574delinsCAACATGCAACATCAT	PM2_Supporting	PVS1	N/A	N/A	N/A	9	Likely pathogenic	Yes (*PMID:**25472526*)
*PRPF31*	c.958delinsGGTTGAGATCGAGCCCA	p.(Leu320GlyfsTer2)	NC_000019.10:g.54128085delinsGGTTGAGATCGAGCCCA	PM2_Supporting	PVS1	N/A	N/A	N/A	9	Likely pathogenic	No
*PRPF31*	c.1015C>T	p.(Gln339Ter)	NC_000019.10:g.54128142C>T	PM2_Supporting	PVS1	N/A	N/A	N/A	9	Likely pathogenic	Yes (PMID: 29957067)
*PRPF31*	c.1190dup	p.(His398ProfsTer77)	NC_000019.10:g.54129100dup	PM2_Supporting	PVS1	N/A	N/A	N/A	9	Likely pathogenic	Yes (PMID: 30718709, 32014492)
*SNRNP200*	c.2041C>T	p.(Arg681Cys)	NC_000002.12:g.96293091G>A	PM2_Supporting + PS4	PP3_Strong	PM1	PP1_Strong	N/A	15	Pathogenic	Yes (PMID: 21618346)
*SNRNP200*	c.2042G>A	p.(Arg681His)	NC_000002.12:g.96293090C>T	PM2_Supporting + PS4	PM5	PM1	N/A	N/A	9	Likely pathogenic	Yes (PMID: 21618346)
*SNRNP200*	c.3260C>T	p.(Ser1087Leu)	NC_000002.12:g.96287968G>A	PM2_Supporting	PP3	PM1_Supporting	PP1_Strong	N/A	7	Likely pathogenic	Yes (PMID: 19878916)

B. Variants where patient has an alternative cause of disease											
Gene	DNA variant	Protein variant	GRCh38 HGVS Genomic coordinate	Population Data	Computational and Predictive Data	Functional Data	Segregation Data	Other Data	Points	Classification	Previously identified
*PRPF3*	c.1345C>T	p.(Arg449Trp)	NC_000001.11:g.150343371C>T	PM2_Supporting	PP3_Strong	PM1_Supporting	N/A	BP5	5	VUS	No
*PRPF8*	c.712C>T	p.(Leu238Phe)	NC_000017.11:g.1681632G>A	PM2_Supporting	PP3	PP2	N/A	BP5	2	VUS	No
*PRPF8*	c.3317C>A	p.(Ala1106Asp)	NC_000017.11:g.1673875G>T	PM2_Supporting	PP3	PP2	N/A	BP5	2	VUS	Yes – on population databases only
*PRPF31*	c.410G>C	p.(Arg137Pro)	NC_000019.10:g.54122584G>C	PM2_Supporting	PP3_Moderate	PP2	N/A	BP5	3	VUS	No
*PRPF31*	c.1114C>T	p.(Arg372Trp)	NC_000019.10:g.54128345C>T	PM2_Supporting	PP3_Moderate	PP2	N/A	BP5	3	VUS	Yes – on population databases only
*SNRNP200*	c.1787T>C	p.(Ile596Thr)	NC_000002.12:g.96295543A>G	PM2_Supporting	PP3	PM1_Supporting	N/A	BP5	2	VUS	No
*SNRNP200*	c.2182C>T	p.(Arg728Trp)	NC_000002.12:g.96291879G>A	PM2_Supporting	PP3_Moderate	PM1_Supporting	N/A	BP5	3	VUS	Yes – on population databases only
*PRPF6*	c.2186G>A	p.(Arg729Gln)	NC_000020.11:g.64027139G>A	PM2_Supporting	PM5	PP2	N/A	BP5	3	VUS	Yes – on population databases only

C. Variants classified as a VUS											
Gene	DNA variant	Protein	GRCh38 HGVS Genomic coordinate	Population Data	Computational and Predictive Data	Functional Data	Segregation Data	Other Data	Points	Classification	Previously identified
*PRPF8*	c.2083G>A	p.(Asp695Asn)	NC_000017.11:g.1677074C>T	PM2_Supporting	PP3_Moderate	PP2	N/A	N/A	4	VUS	No
*PRPF31*	c.1325G>A	p.(Arg442His)	NC_000019.10:g.54129321G>A	PM2_Supporting	PP3	N/A	N/A	N/A	2	VUS	Yes
*SNRNP200*	c.1768C>T	p.(Pro590Ser)	NC_000002.12:g.96295562G>A	PM2_Supporting	PP3_Moderate	PM1_Supporting	N/A	N/A	4	VUS	Yes – on population databases only
*SNRNP200*	c.2578C>A	p.(Gln860Lys)	NC_000002.12:g.96290490G>T	PM2_Supporting	PM5	PM1_Supporting	N/A	N/A	4	VUS	No
*SNRNP200*	c.2990C>G	p.(Thr997Arg)	NC_000002.12:g.96289330G>C	PM2_Supporting	PP3	PM1_Supporting	N/A	N/A	3	VUS	No
*SNRNP200*	c.4708C>T	p.(Arg1570Cys)	NC_000002.12:g.96283590G>A	PM2_Supporting	PP3	PM1_Supporting	N/A	N/A	3	VUS	Yes
*RP9*	c.359G>A	p.(Cys120Tyr)	NC_000007.14:g.33097317C>T	PM2_Supporting	PP3_Strong	N/A	N/A	N/A	5	VUS	Yes – on population databases only
*PRPF6*	c.240+2T>A	p.?	NC_000020.11:g.63983217T>A	PM2_Supporting	PP3	N/A	N/A	N/A	2	VUS	No
*PRPF6*	c.607C>T	p.(Arg203Ter)	NC_000020.11:g.63995084C>T	PM2_Supporting	PM4	N/A	N/A	N/A	3	VUS	Yes – on population databases only

D. Copy-number variants identified in the *PRPF31* gene														
Variant	Gene	Reference	GRCh38 HGVS Genomic coordinate	CNV size	CNV type	Genes included	Section 1 criteria	Section 2 criteria	Section 3 criteria	Section 4 criteria	Section 5 criteria	Total score	Classification	Previously identified
WGD1	*PRPF31*	Hg38	NC_000019.10: g.54106463_54133146del	26kb (26683bp)	Deletion	*NDUFA3*, *TFPT*, *PRPF31*	1A	2C-1	3A	N/A	5B	1	Pathogenic	No (*PMID:**34795310*)
WGD2	*PRPF31*	Hg38	NC_000019.10: g.54036411_54133004del	96kb (96593bp)	Deletion	*VSTM1*, *TARM1*, *OSCAR*, *NDUFA3*, *TFPT*, *PRPF31*	1A	2A	3A	N/A	5D	1.45	Pathogenic	No
WGD3	*PRPF31*	Hg38	NC_000019.10: g.54115410_54131433del	16kb (16023bp)	Deletion	*TFPT*, *PRPF31*	1A	2A	3A	N/A	5F	1	Pathogenic	No
WGD4	*PRPF31*	Hg38	unconfirmed	N/A	Deletion	*PRPF31*	1A	2A	3A	N/A	5F	1	Pathogenic	No

Candidate variants were assigned to 4 sections of Table 1; A. Potential disease-causing candidate variants, B. Variants of uncertain significance (VUS) where the patient has an alternative cause of disease, C. Variants currently classified as a VUS and D. Copy-number variants identified in the *PRPF31* gene. Transcript IDs for each gene are as follows; *PRPF8* = NM_006445.4, *PRPF31* = NM_015629.4, *SNRNP200* = NM_014014.5, *PRPF3* = NM_004698.4, *PRPF6* = NM_012469.4, and *RP9* = NM_203288.2. PMID references are included for previously identified variants. PMID references in italics represent prior publications from this group. Supplemental Table 2 includes evidence for application of ACMG criteria to SNVs in Table 1 part A, B, and C. Supplemental Table 3 includes evidence for application of ACMG criteria applied to CNVs in Table 1 part D. Variant nomenclature has been confirmed using VariantValidator (Supplemental Table 1).

*ACGS*, Association for Clinical Genomic Science; *ACMG*, American College of Medical Genetics and Genomics; *bp*, base pairs; *CNV*, copy-number variant; *N/A*, not applicable; *PMID*, PubMed identifier; *SNV*, single nucleotide variant; *VUS*, variant of uncertain significance; *WGD*, whole gene deletion.

### Variant prioritization and confirmation

Variants were filtered by population allele frequency and in silico pathogenicity scores. Missense variants predicted to be benign were excluded. All frameshift and nonsense variants were analyzed, as were copy-number variants (CNVs) and splice-altering variants with at least 1 SpliceAI score of >0.2. Candidate variants were polymerase-chain-reaction-amplified using 5×FIREPol Master Mix (Cat. no., 04-11-00S25; Solis BioDyne) and Sanger sequenced (Eurofins Genomics) to confirm the presence of variants and to establish phase and segregation of variant(s). Primers used are detailed in Supplemental Table 4.

### Variant interpretation

Pathogenicity of candidate variants was assessed according to American College of Medical Genetics and Genomics (ACMG)/Association for Molecular Pathology, Association for Clinical Genomic Science, and updated ClinGen SVI guidelines.^18^, ^19^, ^20^ Criteria were weighted and numerical scores applied according to ClinGen SVI guidelines. Computational tools used for classification scoring were REVEL,^21^ MetaLR,^22^ M-CAP,^23^ CADD,^24^ AlphaMissense,^25^ and SpliceAI (version 1.3.1).^26^ UniProt (2020_01) was utilized to investigate if variants were located in essential protein domains. Variants in ligand binding domains were assessed for alterations to ligand binding efficiency and affinity using AMDock.^27^ Candidate variants were searched on ClinVar,^28^ PubMed, gnomAD (version 4),^29^ LOVD (version 3.0),^30^ and dbSNP (build 156).^31^ Thresholds for weighting were applied where applicable as per Pejaver et al.^32^ CNVs were interpreted according to ACGS guidelines, CNV classification scoring metrics in ACMG technical standards^33^^,^^34^ and CNV score calculation tool (http://cnvcalc.clinicalgenome.org/cnvcalc/).

### AI-based protein modeling

Variants’ impact(s) on protein structure were modeled using AlphaFold (version 2.3)^35^ and ColabFold (version 1.5.2)^36^ through ChimeraX (version 1.7.1)^37^ and COSMIC-2.^38^ Interactions of proteins resulting from variants with protein partners were modeled using AlphaFold Multimer through COSMIC-2. Hydrogen bonds and inter-residue clashes in a 5Å radius were analyzed using ChimeraX.

### Radar plots

Radar plots were generated after the review of clinical data, which comprised age of onset of adRP, age of onset of cataracts, average BCVA in decimal form, average horizontal visual fields, severity of fundus imaging, color-vision, and rod and cone responses. The scale for the radar plots are described in Supplemental Table 5. The radar plot provides a graphical representation of severity of disease using multiple retinal parameters/features. Each axis represents a different parameter/disease feature. The first (inner) ring in the radar plot signifies the point from which data are plotted. Data for each disease feature were available for most but not all patients. The center point of the radar plot represents the intersection of all axes. A data point at the center point for a specific disease feature/axis indicates that data were unavailable for this disease feature or did not apply. The age at evaluation is available in Supplemental Table 6. Briefly, age of onset and age of onset of cataracts both span ages 0 to 100 at 20 year intervals (0 years = 25, 20 years = 50, 40 years = 75, 60 years = 100, 80 years = 125, 100 years = 150). Average BCVA in decimal format spans 0.1 to 1.25 at intervals of 0.25 decimal VA. Average horizontal visual fields remaining span 25 degrees to 150 degrees at 25-degree intervals. A descriptor was used for severity of fundus images as assigned by the clinical teams and plotted as severe, moderate, mild, or normal. Similarly, color-vision was classified as nonrecordable, diffuse error, tritanopia, or normal. Rod and cone responses were plotted as nonrecordable, reduced and delayed, reduced, or normal.

## Results

**Figure 1:**
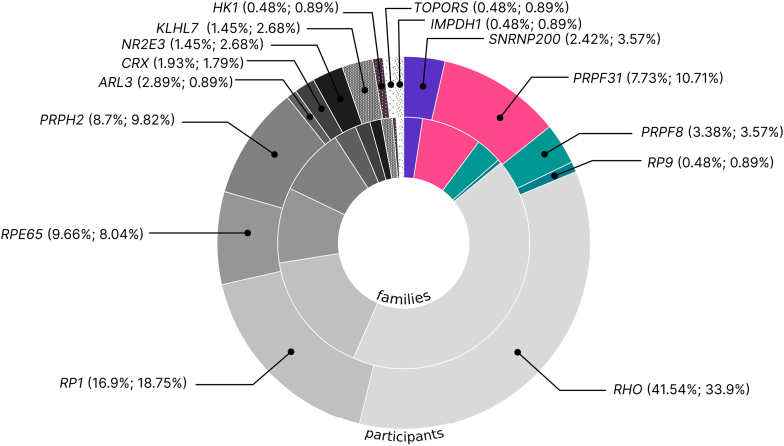
**Genes causative of adRP in theIrishcohort.** The middle ring corresponds to the number of family pedigrees and the outer ring to the number of participants solved by each gene. Percentages are listed as the percentage of participants with adRP with causative variants in the gene followed by the percentage of adRP families associated with the gene. *adRP*, autosomal dominant retinitis pigmentosa.

A total of 635 patients with a clinical diagnosis of RP were screened for inclusion. Of these, 236 had a clinical diagnosis of adRP and 207 had a genetic diagnosis of adRP. Thirty-six variants were identified in *PRPF31*, *PRPF8*, *SNRNP200*, *PRPF3*, *RP9*, and *PRPF6.* Of these, 19 were potential disease-causing candidates classified as likely pathogenic/pathogenic and identified in *PRPF31*, *PRPF8*, and *SNRNP200* (see Table 1A and D, Supplemental Table 2). Several NGS approaches were utilized to identify the variants reported in this study. Of the 45 participants, 23 had target-capture panel sequencing, 10 had exome sequencing, 2 had genome sequencing, 2 had smMIPs and finally, 8 participants had targeted Sanger sequencing to confirm a previously identified familial variant (Supplemental Table 7). This represents 14% of individuals (18.7% of pedigrees) with adRP with a known genetic cause in the cohort (Figure 1). 17 variants identified in the genes *PRPF3*, *PRPF8*, *PRPF31*, *SNRNP200*, *PRPF6*, and *RP9* were classified as variants of uncertain significance (VUS) of which 8 variants were identified in cases with an alternative cause of disease (Table 1B and C). Candidate variants in *CWC27*, *DHX38*, *PRPF4*, *PRPF38A*, and *USH1G* were not detected. Thirteen of the 36 variants identified were absent from PubMed, gnomAD (version 4), LOVD and ClinVar, whereas 10 of the 36 variants had not previously been associated with IRDs. This study includes 45 individuals with RP from 34 families (Supplemental Figure 1). The mutational spectrum consisted of 22 missense, 2 splice-altering, 5 frameshift, 1 single in-frame codon deletion, and 2 nonsense variants (Table 1, Supplemental Table 1). Four heterozygous CNVs involving complete deletion of *PRPF31* were identified (Table 1D, Supplemental Table 3). Although most cases were autosomal dominant, 1 was autosomal recessive because of a homozygous variant in *SNRNP200*. All individuals presented with severe and progressive RP. The category severe corresponds to the legal classification for visual impairment based on the degree of visual field loss and/or the decrease in visual acuity. However, those with variants in PRPF genes were more severely affected than those with variants in *SNRNP200*. This study provides an updated view of the genes that are causative of adRP in the Irish population (Figure 1).

### PRPF8

3.38% of adRP cases (3.57% solved adRP families) were attributable to *PRPF8* variants. PRP8, encoded by *PRPF8*, is the most highly conserved spliceosome protein, with a missense constraint score of *z* = 11.324. PRP8 is key in organizing the catalytic core by forming the U5 snRNP, regulating SNRNP200 and facilitating pre-mRNA splicing.^39^ We identified 7 variants, 6 of which were missense affecting highly conserved amino acids. Two variants were absent from PubMed, gnomAD, LOVD, and ClinVar.

**Figure 2:**
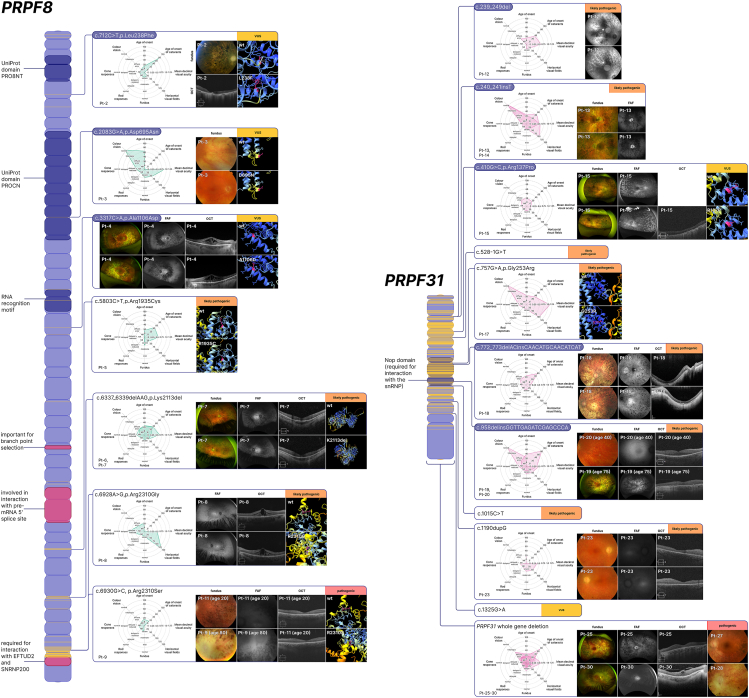
**Distribution of *PRPF8* and *PRPF31* variants.** Schematic for each gene (lilac), protein domains (dark purple), protein-binding domains (pink), and variants (yellow bar). For variants identified in the study, clinical impact is denoted by a radar plot which represents the age of onset of RP, age of onset of cataracts, mean decimal visual acuity, horizontal visual fields, severity of fundus changes, rod and cone responses, and color-vision plotted to scale (Supplemental Table 5). For patients with severe disease, the color bar in the radar plot is more constricted when compared with those with milder disease. Clinical imaging is shown for each variant and patient where possible, including color fundus, fundus autofluorescence, and OCT images. Variant classifications and AlphaFold models are presented. *RP*, retinitis pigmentosa; *OCT*, optical coherence tomography.

All variants classified as likely pathogenic lay toward the C-terminal MPN domain required for interaction with EFTUD2 and SNRNP200.^40^ c.6337_6339del p.(Lys2133del) is highly conserved (Supplemental Figure 2), classified as likely pathogenic, and previously published by this group and others.^41^ AlphaFold models predict gross alterations to protein structure and loss of hydrogen bonds in this region (Figure 2). Given that the reading frame remains intact, this suggests that the Lys2133 residue may be critical for PRP8/EFTUD2/SNRNP200 interactions or folding kinetics of PRP8. Clinical features included nyctalopia in teens, progressing to absence of rod responses by age 30 (Figure 2, Pt-6 and 7). Two variants affected the highly conserved Arg2310 residue (Figure 2). AlphaFold Multimer predictions suggest that c.6928A>G p.(Arg2310Gly) (Pt-8) and c.6930G>C p.(Arg2310Ser) (Pt-9-11) alter PRP8 binding to EFTUD2 and SNRNP200 (Supplemental Figure 3). These variants resulted in severe RP, with onset as young as 10 years (Figure 2, Supplemental Table 6).

Residue Arg1935 in the PRP8 RNase H homology domain appears to be of significant importance, with variant c.5803C>T p.(Arg1935Cys) (Pt-5) and p.(Arg1935His) (identified in another study) classified as likely pathogenic.^42^

Three VUS were identified toward the N-terminal of the protein, in the reverse transcriptase homology domain: c.712C>T p.(Leu238Phe) (Pt-2), c.2083G>A p.(Asp695Asn) (Pt-3), and c.3317C>A p.(Ala1106Asp) (Pt-4). In silico pathogenicity prediction tools indicated likely damaging effects. AlphaFold modeling predicted several clashes and alterations to hydrogen bonds within the protein resulting from variants p.(Leu238Phe) and p.(Ala1106Asp), whereas p.(Asp695Asn) was not predicted to have a meaningful impact on protein structure (Figure 2). Of note, the phenotypes associated with c.712C>T p.(Leu238Phe) (Pt-2) and c.2083G>A p.(Asp695Asn) (Pt-3) were vastly different despite being in the same domain. Pt-3 manifested symptoms at 60 with BCVA of 6/7.5; however, rod responses were nonrecordable, cone responses were delayed and reduced in amplitude and visual fields were concentrically constricted to <40° fixation (Supplemental Table 6). In contrast, age of onset of Pt-2 was 8 and at age 68, vision had deteriorated to perception of light (PL).

### PRPF31

*PRPF31* variants were the fifth most common cause of adRP after variants in *RHO*, *RP1*, *PRPH2*, and *RPE65* accounting for 7.73% of cases and 10.71% of families (Figure 1). Fifteen variants were identified, of which 3 were absent from PubMed, gnomAD, LOVD, and ClinVar highlighting the diversity of variants causative of *PRPF31*-linked adRP. Incomplete penetrance was observed in several families, including those with whole gene deletions (family T). PRP31 is a subunit of U4 snRNP and participates in U4/U6.U5 tri-snRNP complex formation through interaction with PRP6.^39^ Several variants (c.757G>A p.Gly253Arg, c.772_773delinsCAACATGCAACATCAT, and c.958delinsGGTTGAGATCGAGCCCA), of which the latter 2 are out of frame, lie in the highly conserved Nop domain involved in interaction with U4 snRNP, in which there are no reported benign missense variants. Protein models indicate that c.757G>A p.(Gly253Arg) may alter overall protein structure, resulting in clashes with neighboring residues within the U4 snRNP-interacting region. The other variants identified in this domain are predicted to result in nonsense-mediated decay and were therefore not modeled.

Missense variants outside of the Nop domain were classified as VUS. c.410G>C p.(Arg137Pro) (Pt-15, Figure 2), located between the 2 coiled domains, is predicted to be disruptive with protein models indicating loss of local hydrogen bonds and altered tertiary structure. Similarly, in silico tools indicate potentially damaging effects of c.1114C>T p.(Arg372Trp) (Pt-22). However, these individuals have clinical diagnoses of Bardet-Biedl syndrome and choroideremia respectively, downgrading these variants to tepid VUS, albeit these variants may be contributing to retinal pathology. Cool VUS c.1325G>A p.(Arg442His) (Pt-24) is not associated with a conserved functional domain nor is it predicted to affect protein structure (Figure 2). VUS c.1114C>T p.(Arg372Trp) and c.1325G>A p.(Arg442His) were observed 22 and 37 times in gnomAD, respectively; however, incomplete penetrance is a feature of *PRPF31*-linked adRP.

The majority of variants identified in *PRPF31* function via a LOF mechanism, resulting in truncated transcripts predicted to undergo nonsense-mediated decay (Table 1, Supplemental Table 1). Four pathogenic CNVs were identified resulting in a whole gene deletion of *PRPF31* (Pt-25-30, Figure 2, Table 1D, Supplemental Table 3). Clinical phenotypes were typical of *PRPF31*-related adRP (Supplemental Table 6), with symptoms commencing as early as the first decade. Some individuals maintained relatively good central vision, albeit concentrically constricted to <5° fixation. Rod and cone responses were typically nonrecordable by age 50 in participants with *PRPF31* variants. Several individuals presented with tritanopia but without tritanopia-associated variants in *OPN1SW.*

### SNRNP200

*SNRNP200*-related adRP accounted for 2.42% of cases and 3.57% of solved adRP families in this cohort compared with 1.1% to 1.6% of cases elsewhere (Figure 1).^43^ SNRNP200 facilitates unwinding of the U4/U6 snRNA duplex in the activation of the precatalytic spliceosome.^8^ We detected 9 missense variants: 3 were absent from PubMed, gnomAD, LOVD, and ClinVar, whereas 2 variants were not previously reported in relation to IRDs.

**Figure 3:**
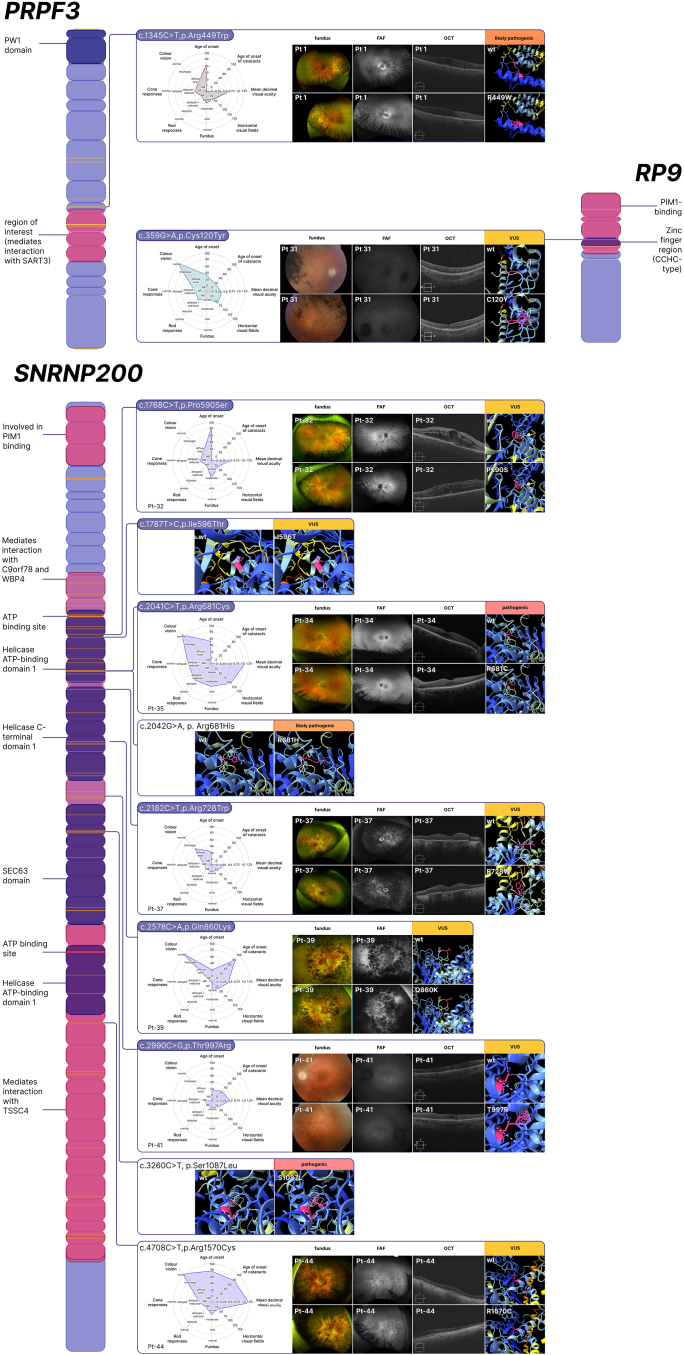
**Distribution of each variant in the *PRPF3*, *SNRNP200*, and *RP9* genes.** Schematic for each gene (lilac), protein domains (dark purple), protein-binding domains (pink), and variants (yellow bar). For variants identified in the study, clinical impact is denoted by a radar plot, which represents the age of onset of RP, age of onset of cataracts, mean decimal visual acuity, horizontal visual fields, severity of fundus changes, rod and cone responses, and color-vision plotted to scale (Supplemental Table 5). For patients with severe disease, the color bar in the radar plot is more constricted when compared with those with milder disease. Clinical imaging is shown for each variant and patient where possible, including color fundus, fundas autofluorescence and OCT images. Variant classifications and AlphaFold models are presented. *RP*, retinitis pigmentosa; *OCT*, optical coherence tomography.

The variants c.1768C>T p.(Pro590Ser) (Pt-32) and c.1787T>C p.(Ile596Thr) reside in the helicase ATP-binding domain, which facilitates separation of double-stranded nucleic acids. c.1768C>T p.(Pro590Ser) is a warm VUS, and although protein modeling suggests that it does not affect protein structure, in silico tools predict a damaging effect with moderate evidence (Figure 3, Table 1). c.1787T>C p.(Ile596Thr) is a cool VUS observed in 9 individuals in the Target 5000 cohort, many of whom have alternative clinical and genetic diagnoses. Pathogenic variants c.2041C>T p.(Arg681Cys) (Pt-35) and c.2042C>A p.(Arg681His) (Pt-36) reside in a region interacting with C9orf78 and WBP4.^44^ Protein modeling predicts that both missense variants disrupt local hydrogen bonding and p.(Arg681His) introduces clashes with neighboring residues (Figure 3). Although protein structure immediately surrounding the variants is predicted to remain broadly unchanged from wild-type, when complexed with C9orf78 or WBP4, overall quaternary structure is disrupted, suggesting that small changes locally may result in a butterfly effect across the protein (Supplemental Figure 4). Analyses of clashes within predicted complexes corroborates this for p.Arg681Cys resulting in 629 clashes relative to 429 for wild-type SNRNP200 when complexed with C9orf78, and 909 relative to 473 for wild-type when complexed with WBP4. However, there were fewer predicted changes in clashes for the p.Arg681His variant with 352 clashes versus 428 when complexed with C9orf78 and 531 clashes versus 473 when complexed with WBP4. Of note, these are predictive models and as yet are not used to inform variant classification via ACMG guidelines. Variants c.2182C>T p.(Arg728Trp) (Pt-37) and c.2578C>A p.(Gln860Lys) (Pt-39) are in the helicase C-terminal domain, which binds ATP and stimulates the N-terminal helicase^45^ and have been classified as tepid and warm VUS, respectively. Pt-37 has a clinical and genetic diagnosis of Usher Syndrome, and c.2182C>T p.(Arg728Trp) has also been observed in an unaffected family member, suggesting that it may be benign. Interestingly, protein modeling and in silico pathogenicity predictions suggest p.(Arg728Trp) is more deleterious than p.(Gln860Lys) (Figure 3, Table 1). In silico molecular docking analyses suggest neither variant affects ATP-binding capacity.

Variants c.2990C>G p.(Thr997Arg) (Pt-41) and c.3260C>T p.(Ser1087Leu) (Pt-43) are located in the SEC631 domain, which facilitates C9orf78 and WBP4 interactions.^44^ c.2990C>G p.(Thr997Arg) is classified as a tepid VUS. Interestingly, Pt-41 is homozygous, whereas their mother is heterozygous and thus far unaffected. The macula is relatively well preserved in Pt-41; however, rod and cone responses were extinguished by age 19 (Figure 3, Supplemental Table 6). Protein models reveal dramatically reduced confidence in folding kinetics, and increased clashes compared with wild type (Supplemental Figure 4). c.3260C>T p.(Ser1087Leu) is an established likely pathogenic variant; however, protein models do not appear as severely altered as p.(Thr997Arg), with interactions with neighboring proteins predicted to be unaffected. Lastly, warm VUS c.4708C>T p.(Arg1570Cys) in the helicase C-terminal 2 domain in Pt-44 did not present with symptoms until their 70s (Figure 3, Supplemental Table 6) and is inherited by their child who remains unaffected at age 50. Additionally, there are 30 heterozygotes in gnomAD and the protein model remains largely unaltered; given the above and that incomplete penetrance is a feature of *SNRNP200*, this variant remains a VUS.

### PRPF3

Pt-1 represents the only case in our cohort with a candidate variant in *PRPF3*. PRP3 binds the U4/U6 snRNA duplex and plays a role in tri-snRNP stability.^39^ Hot VUS c.1345C>T p.(Arg449Trp) is in a conserved region that interacts with SART3-required for U4/U6 snRNP recycling-and RP9, also associated with adRP.^46^ Modeling suggests that c.1345C>T p.(Arg449Trp) alters overall protein structure and interaction with PRP4 and SART3 (Figure 3, Supplemental Figure 5). Although wild-type PRP3 protein modeled with SART3 had 369 unfavorable inter- and intraprotein clashes, Arg449Trp PRP3 modeled with SART3 had considerably more clashes at 458. Alterations to PRP3 and PRP4 binding were more subtle, with the Arg449Trp protein resulting in a more constricted complex (Supplemental Figure 5). Of note, Pt-1 appears to exhibit dual genetic diagnosis (DGD) accounting for adRP, with co-occurrence of a pathogenic variant in *RP1*.

### RP9

adRP caused by *RP9* variants is particularly rare with only 1 incidence detected in this cohort (Pt-31) and just 3 missense variants documented to date. Although the function of RP9 is still unclear, it is known to function as a splicing factor and bind to PRP3.^39^ Variant c.359G>A p.(Cys120Tyr), classified as a hot VUS, is within the zinc-finger and PIM1-binding region (Figure 3). Despite a conservative amino acid change, in silico modeling predicts gross alterations to protein structure and multiple clashes with neighboring residues. Moreover, protein modeling of RP9 complexed with PRP3 predicts gross changes to quaternary structure and additional clashes with neighboring residues (Supplemental Figure 6). Pt-31 was diagnosed age 20 and experienced progressive loss of visual fields to <50° fixation at age 35. Both rod and cone responses are significantly delayed and reduced in amplitude (Figure 3, Supplemental Table 3).

### PRPF6

*PRPF6*-associated adRP accounted for 0% of cases in our study and other large adRP cohorts^13^^,^^47^ but has been associated with approximately 2% of cases in Han Chinese populations.^48^ Tepid VUS c.2186G>A p.(Arg729Gln) was detected in a participant with a clinical and genetic diagnosis of Best disease. The highly conserved Arg729 lies in the C-terminal tetratricopeptide repeat HAT 8 domain, which interacts with U4/U6 snRNP. PRP6 also associates with PRP31 and coparticipates in the formation of the tri-snRNP complex.^39^ Established pathogenic variant p.(Arg729Trp) affects the same residue as variant p.(Arg729Gln) but is a nonconservative amino acid change, compared with the conservative missense variant identified here. Additionally, in silico tools and protein models do not suggest a deleterious impact of p.(Arg729Gln) (Table 1, Supplemental Figure 7).

## Discussion

Perturbation of RNA processing has been implicated in a diverse range of disorders, including IRDs. Herein, the landscape of candidate disease-causing variants in pre-mRNA-processing genes in IRDs has been characterized in an Irish RP cohort. Variants in pre-mRNA-processing genes account for 14% of solved adRP cases in this patient cohort. Notably, 13 variants were absent from PubMed, gnomAD, LOVD, and ClinVar, whereas a further 10 variants found in population databases were not previously associated with an IRD. Thus, together these variants account for almost ∼60% of variants found highlighting the mutational diversity in RNA processing-linked IRD genes. The study included 45 individuals with RP from 34 unrelated families. Variants in these genes have been associated with 15% to 20% of adRP cases in other populations, consistent with our findings.^3^ Cumulatively, variants in pre-mRNA-processing genes are a common cause of adRP in Ireland second to *RHO* variants accounting for 41.5% of cases (33.9% of families); Figure 1. Notably, variants in *RPE65* represent 9.6% of cases (8.0% of families) because of the well-established dominant variant c.1430A>G p.(Asp477Gly).^49^

Noncoding small nuclear RNAs are critical components of the spliceosome and have garnered attention because of their implication in disease, such as the U4 snRNA gene (*RNU4-2*) with neurodevelopmental disorders.^50^ Until recently, RP-associated variants have been restricted to U4/U6.U5 tri-snRNP proteins. However, Quinodoz et al^7^ have detected variants in *RNU4-2* and four RNU6 paralogs, which cause 1.2% of the undiagnosed RP cases in their cohort highlighting the role of snRNAs in monogenic diseases.

The inheritance pattern of variants in pre-mRNA-processing genes is primarily autosomal dominant; however, autosomal recessive variants have been reported in *SNRNP200*.^51^ Notably, the phenotype of Pt-41 harboring the homozygous *SNRNP200* c.2990C>G p.(Thr997Arg) variant is consistent with that of family B harboring biallelic variants in *SNRNP200* described previously by Gerth-Kahlert and Koller.^51^ Pt-41 in this study has a ring of hyperautofluorescence at the macula. Furthermore, pt-41 lacks the hypoautofluorescence in the peripheral retina, which tends to be observed in dominant cases as described previously.^51^ Additionally, incomplete penetrance has been observed in families with *PRPF31*-related adRP and to a lesser extent in *PRPF8*, *SNRNP200*, *RP9*, and *PRPF6*.^12^^,^^52^, ^53^, ^54^ Incomplete penetrance renders the interpretation of variants challenging. For example, the criterion BS2 “observations in controls” must be applied with caution because of incomplete penetrance. Additionally, the maximum tolerated allele frequency in gnomAD may be too restrictive for genes exhibiting incomplete penetrance, as noted for several variants in this study (Supplemental Table 2). Similarly, when tracking segregation of a variant, there may be asymptomatic heterozygotes as is the case in families T, C2, and G2 (Supplemental Figure 1). This poses a risk that unaffected heterozygotes could be mistaken for BS4, “non-segregation with disease.”^19^ Therefore, caution should be taken when classifying variants in these genes.

The majority of candidate variants identified in *PRPF31* are LOF; however, 4 variants are missense, of which 3 are classified as VUS. Of note, c.757G>A p.(Gly253Arg), was classified as likely pathogenic and may cause adRP by reducing PRP31’s affinity to U4 snRNP. Additionally, 4 CNVs of different sizes involving complete deletion of *PRPF31* were identified and classified as pathogenic, supporting haploinsufficiency as a disease mechanism (Table 1, Supplemental Table 3). Frameshift, nonsense, and missense variants can cause *PRPF8* and *SNRNP200*-related adRP according to the literature; however, missense variants are more common. Likewise, almost all disease-causing variants in *PRPF3* are missense and reside in the C-terminal conserved region. Variants in splicing factor *RP9* represent a rare cause of adRP with just 1 individual detected in our cohort and only 3 other missense variants documented to date. In agreement with Wang et al,^13^ *PRPF6* LOF variants should be interpreted with caution given that the frequency of LOF variants in gnomAD is greater than the incidence of adRP and the probability of loss-of-function intolerance (pLI) for this gene is 0. Therefore, the 2 LOF variants identified here are likely not causative of disease.

Table 1A and D separates the candidate variants we believe are causative of disease from the variants whose role in disease is uncertain in Table 1B and C. In total, we identified 19 candidate variants, including 4 whole gene deletions which are very likely causative of disease. Seventeen additional variants have been included because of variant type, in silico pathogenicity scores, and rarity in population databases (Table 1B and C). However, because of predicted disease mechanism(s) and presence in cases with alternative genetic causes, these 17 variants remain VUS and therefore require additional evidence to upgrade/downgrade their classification.

Genotype-phenotype studies suggest that nonsense, frameshift, missense, and indels in *PRPF31* could display dominant negative effects as age of onset tends to be earlier compared with large deletions.^11^ However, in contrast, Pt-30 with a 16-Kb whole-gene deletion had the earliest age of onset (7 years), Pt-26 and Pt-27 with a 96-Kb deletion developed symptoms at ages 16 and 22, respectively, and finally, Pt-25 with a 26-kb deletion manifested symptoms at age 17. Age of onset for other *PRPF31* variant types were between 17 and 34. Of note, all participants with *PRPF31*-RP typically presented with night vision deficits that progressed to loss of peripheral vision (Figure 2, Supplemental Table 6). All affected individuals had nonrecordable rod and cone responses and concentrically constricted visual fields. Individuals with *PRPF8*-related adRP also had a severe phenotype, perhaps because of its multimodal functions in pre-mRNA-processing. However, Pt-3 had a milder form of RP and given current evidence regarding the c.2083G>A p.(Asp695Asn) variant, this VUS may be hypomorphic or, indeed, not causative in this case. Of note, several variants identified elsewhere, such as p.(Pro12Leu) and p.(Met25Thr) in the N-terminal of PRP8, are associated with primary open angle glaucoma.^55^ Interestingly, Pt-2, with variant c.712C>T p.(Leu238Phe) located in the N terminus had glaucoma in addition to a severe and complex adRP phenotype. In family (A), the proband and daughter are affected and both are heterozygous for an established pathogenic variant in *RP1* (NM_006269.2:c.2613dupA p.(Arg872Thrfs∗2)). Interestingly, their phenotypes are quite different which may be explained by co-occurrence of the *PRPF3* c.1345C>T p.(Arg449Trp) in Pt-1 only (Figure 3, Supplemental Table 6). Pt-1 does show characteristic features of *RP1*-associated adRP. Therefore, Pt-1 could exhibit DGD or the *PRPF3* variant may play a minor role in this condition. Based on the clinical data available, participants with *SNRNP200* variants typically presented with a milder phenotype compared with *PRPF*-related adRP. First symptoms include night vision and visual field defects followed by moderate changes to fundus, nonrecordable/reduced rod responses and cone responses that are further reduced and delayed in amplitude as the condition progresses. However, those with *SNRNP200* variants retained better visual acuity. This has been recently reported in a longitudinal study of patients with *SNRNP200*-associated RP, which similarly found central retinal function was preserved until the 6th decade of life, indicating that *SNRNP200*-related cases may be amenable to gene therapy interventions.^56^ One could potentially hypothesize that marked differences in RP severity between PRPF and SNRNP200-related cases may be due to the different functions these proteins exert in pre-mRNA splicing. For example, the PRPF genes are essential components of the U4/U6.U5 tri-snRNP complex, whereas *SNRNP200*’s encoded protein is involved mainly in the assembly and disassembly of the spliceosome. This process may be redundant, and/or variants in the Brr domains of SNRNP200 required for spliceosome assembly and disassembly may be hypomorphic. Interestingly, Pt-33 and Pt-35 from different pedigrees harbor the same c.2041C>T p.(Arg681Cys) variant but ages of onset were 46 and 5, respectively, highlighting the clinical heterogeneity evident in adRP.

Interestingly, although many individuals had color vision impairment with no distinct pattern, several had tritanopia, of whom all but 1 had a variant in *PRPF31*, and no variants were detected in *OPN1SW* to indicate a congenital blue-yellow colour defect. Tritanopia is the most commonly reported colour-vision defect in individuals with advanced RP.^57^ Evidence suggests that blue-light-sensitive S-cones are more vulnerable than M- and L-cones, with significantly greater loss of S-cone responses in participants with RP.^58^ S-cones are located within the perifovea and periphery of the retina, the region severely affected by RP. Given that tritanopia is associated with advanced RP, the frequency of this defect within the *PRPF31* cohort underscores the severity of *PRPF31-*adRP.

Of note, 8 individuals in this study (Pt-1, 2, 4, 15, 22, 37, 38, and 45) presented with 2 possible genetic diagnoses (Supplemental Figure 1). Although rare, this phenomenon of DGD has been observed previously given the large-scale genetic heterogeneity in IRD genes. In a study by Daiger et al,^59^ of 300 confirmed adRP cases, 1% had multiple segregating variants. Because of the incidence of DGD in our cohort and others, it may be advisable to use BP5 “found in a case with an alternative cause” with caution, otherwise, it could downgrade a likely pathogenic variant to a VUS.^19^ This situation was encountered when interpreting the *PRPF3* variant in Pt-1. Further evidence in support of this variant’s pathogenicity, such as the known pathogenic variant *PRPF3* p.(Arg449Gly) reported by Zhong and Yan,^60^ could not be used to upgrade this variant because PP3_Strong was already applied constituting a stronger piece of evidence within the computational and predictive category, and only 1 piece of evidence is allowed per category as per ACMG/Association for Molecular Pathology guidelines. The double counting of 2 pieces of evidence within one category is deemed to potentially lead to misdiagnosis, incomplete diagnosis, and inaccurate recurrence risk estimates.

This study presents extensive variant interpretation alongside protein modeling. Notably, there is a high degree of concordance between variant classifications and the predicted impact of these variants on the resultant protein. For instance, *SNRNP200* p.(Ile596Thr) does not appear to affect protein structure and is classified as a cool VUS despite relatively high in silico pathogenicity prediction scores. In comparison, *SNRNP200* variants p.(Arg681Cys) and p.(Arg681His) in domains involved in interaction with C9orf78 and WBP4 were classified as pathogenic and likely pathogenic, corroborated by AlphaFold findings that reveal alterations to gross quaternary structure when *SNRNP200* is complexed with either protein partner.

The development of therapies targeting retinal spliceopathies will be advanced through greater understanding of the effects of disease variants. RNAseq from retinal cells obtained from patient-derived iPSCs is likely to shed light on the degree of splicing perturbation, including which retinal genes are subject to the greatest splicing alterations. This will inform potential therapeutic developments but also provide insights into why the retina is often exclusively affected by variants in these genes.

Pre-mRNA-processing is essential in all eukaryotic cells. This study characterizes the genetic landscape of variants in genes encoding pre-mRNA-processing factors in an Irish RP cohort providing in-depth classifications and documenting corresponding phenotypes. Moreover, variants in this group of genes account for 14% of solved adRP cases in our cohort. Of note ∼60% of the variants found were either absent from databases PubMed, gnomAD, LOVD, and ClinVar or not previously associated with an IRD, highlighting the mutational diversity in RNA processing-linked IRD genes. Indeed, these findings in combination with the phenotypic spectrum observed significantly add to the elucidation of the genetic architecture of pre-mRNA-processing factors implicated in RP and aid the interpretation of variants in these genes for future patients. Given the severity of disease and the autosomal dominant inheritance pattern, investigating such genotype-phenotype correlations and compiling the evidence to reclassify VUS is essential to provide patients with an accurate prognosis and optimal care pathways.

## Data Availability

The original contributions presented in the study are included in the article/Supplemental Material. Further inquiries can be directed to the corresponding author. Supplemental Figures 1 Pedigrees; Supplemental Figures 2-7 AlphaFold protein models; Supplemental Table 1 Variant nomenclature validated using VariantValidator; Supplemental Table 2 Single Nucleotide Variant interpretation; Supplemental Table 3 Copy Number Variant interpretation; Supplemental Table 4 Primers used; Supplemental Table 5 Radar plot scale; Supplemental Table 6 Clinical data; Supplemental Table 7 Type of NGS methodology employed.

## Conflict of Interest

The authors declare no conflicts of interest.
