# Supplementary material for: The landscape of variants in pre-mRNA-processing factor genes in an Irish cohort
Source: Genet Med Open. 2026 May 6;4:104402. doi: 10.1016/j.gimo.2026.104402 (PMC13332460; doi:10.1016/j.gimo.2026.104402)
Supplement: Supplemental Figure 1 [file mmc1.pdf]

Pedigree trees for families A-H2

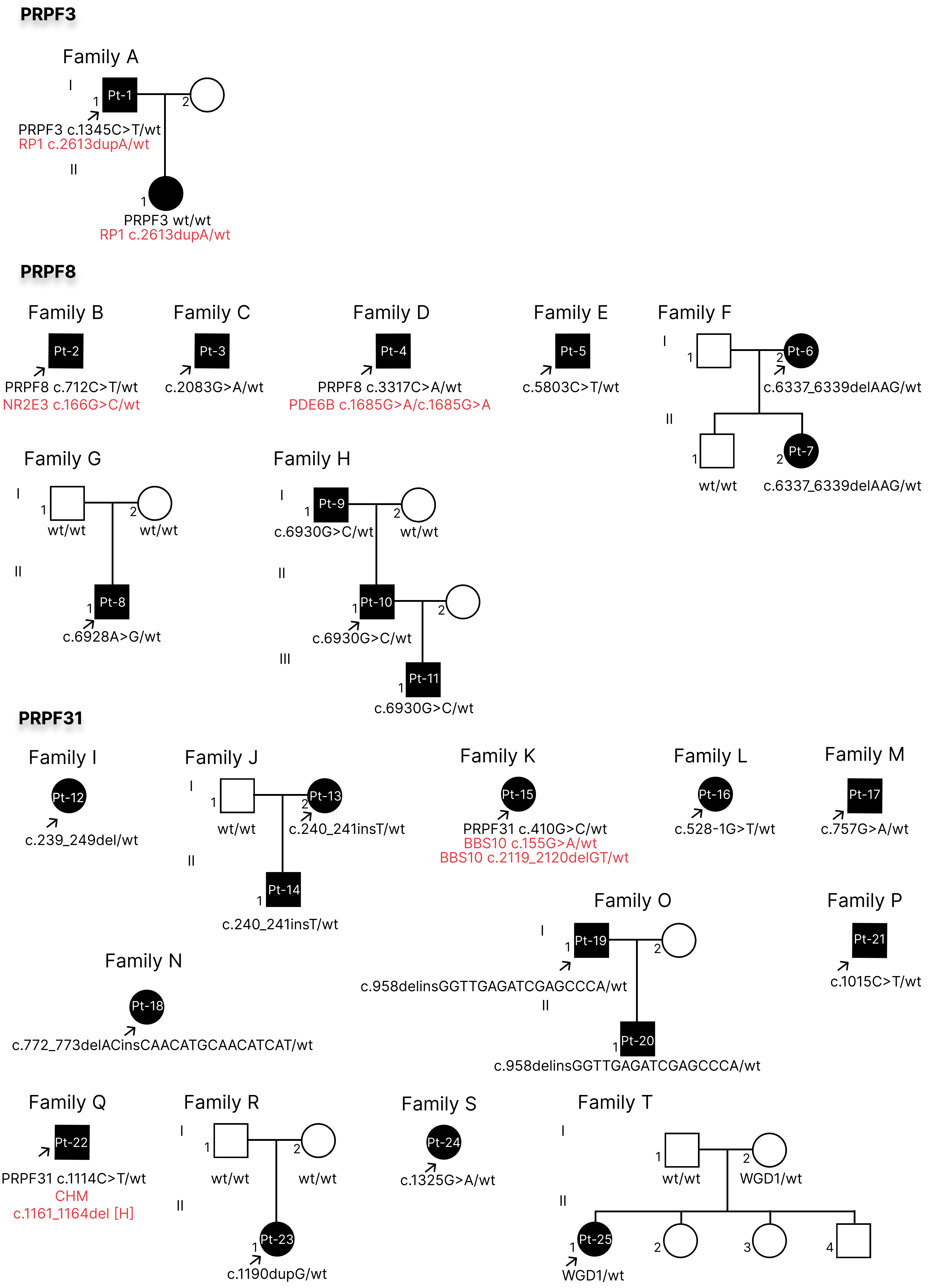

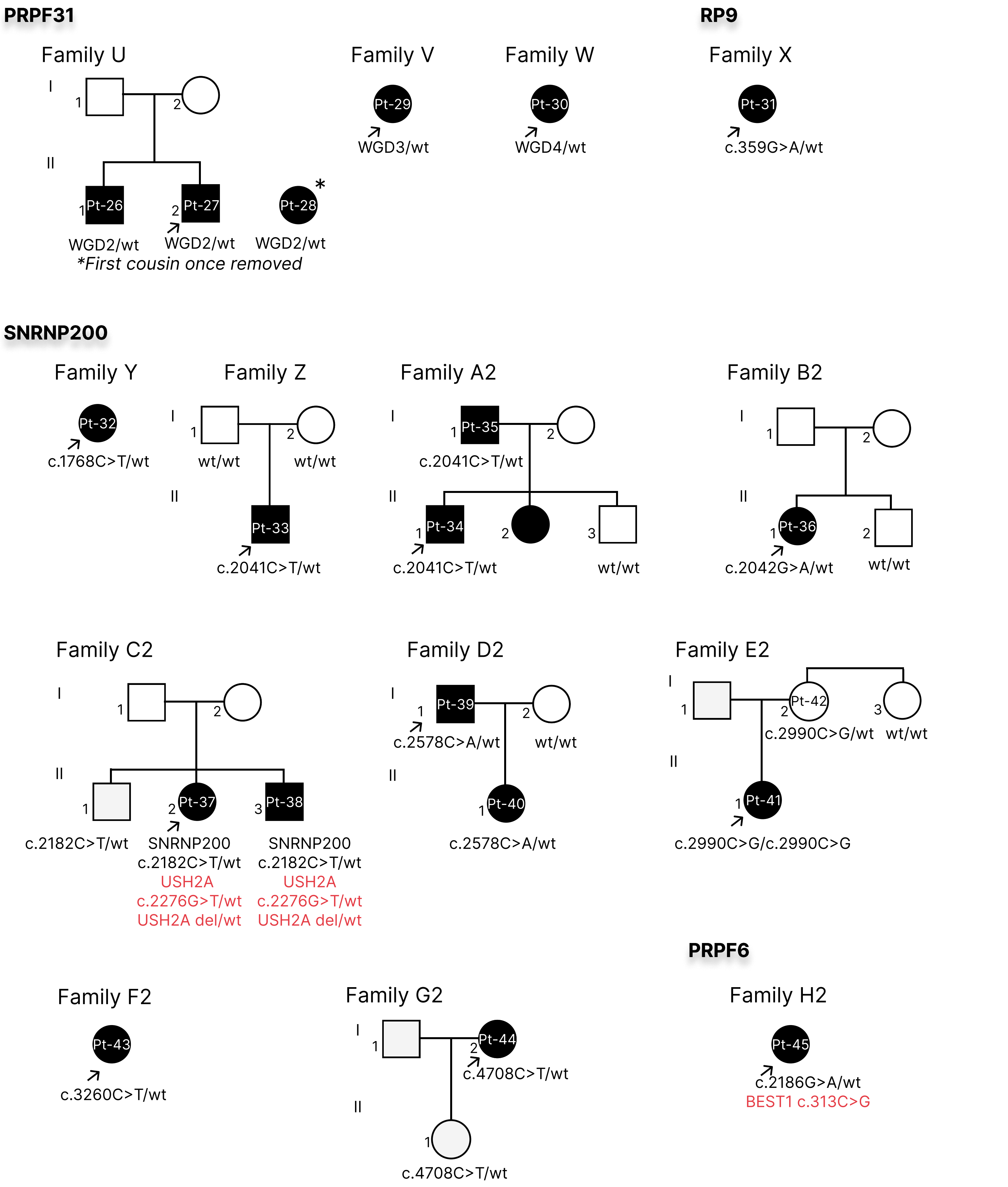

Supplementary figure 1 Pedigree trees for Family A-H2. Arrows indicate the probands in each family. Affected males are denoted by the shaded squares and affected females are denoted by the shaded circles. Variants are illustrated below each individual with wt referring to the wild-type allele. Variants written in red text indicate additional variants causative of disease. [H] refers to individuals who are homozygous for a given variant.
