# Supplementary material for: The landscape of variants in pre-mRNA-processing factor genes in an Irish cohort
Source: Genet Med Open. 2026 May 6;4:104402. doi: 10.1016/j.gimo.2026.104402 (PMC13332460; doi:10.1016/j.gimo.2026.104402)
Supplement: Supplemental Figures 2-7 [file mmc2.docx]

**Supplementary Figures 2-7**


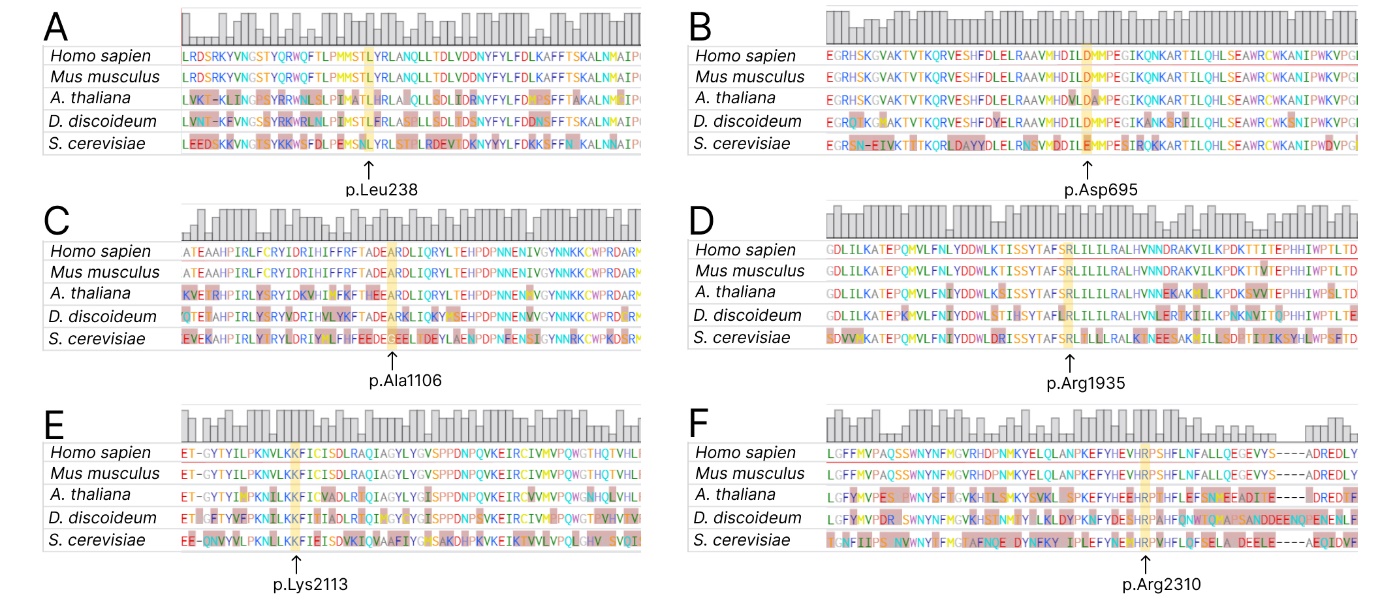


**Figure 2. PRP8 conservation across species.** A conservation Leucine at position 238 of the PRP8 protein, B conservation of Aspartic acid at position 695, C conservation of alanine at position 1106, D conservation of Arginine at position 1935, E conservation of Lysine at position 2113 and F conservation of Arginine at position 2310.

**
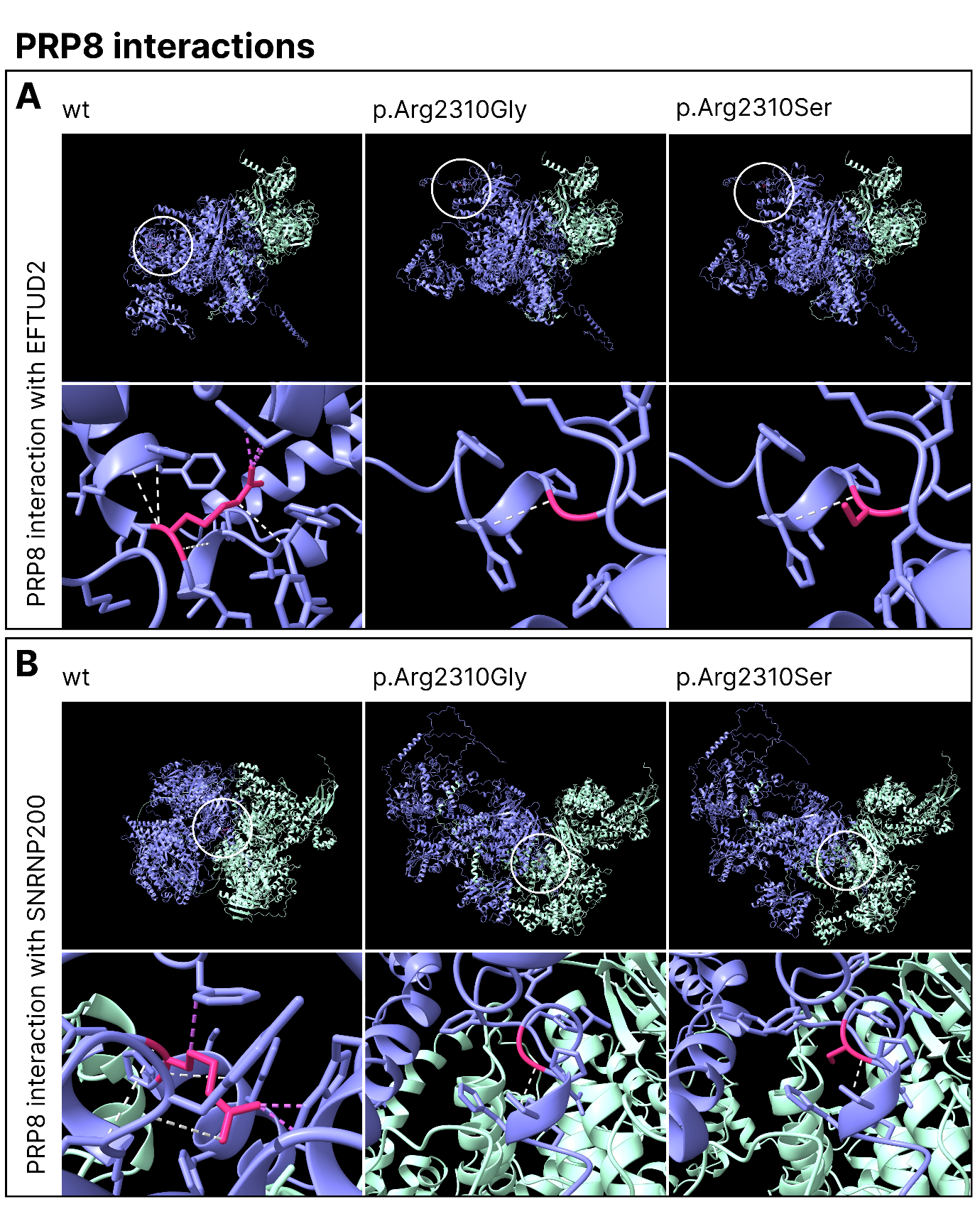
Figure 3. PRP8 AlphaFold multimer modelling.** A interaction of wildtype (WT) and variant (p.Arg2310Gly and p.Arg2310Ser) PRP8 with EFTUD2 protein partner. B interaction of wildtype (WT) and variant (p.Arg2310Gly and p.Arg2310Ser) PRP8 with SNRNP200 protein. The residue of interest is coloured in pink. Hydrogen bonds are denoted by the white dashed lines and clashes are denoted by the purple dashed lines.

**
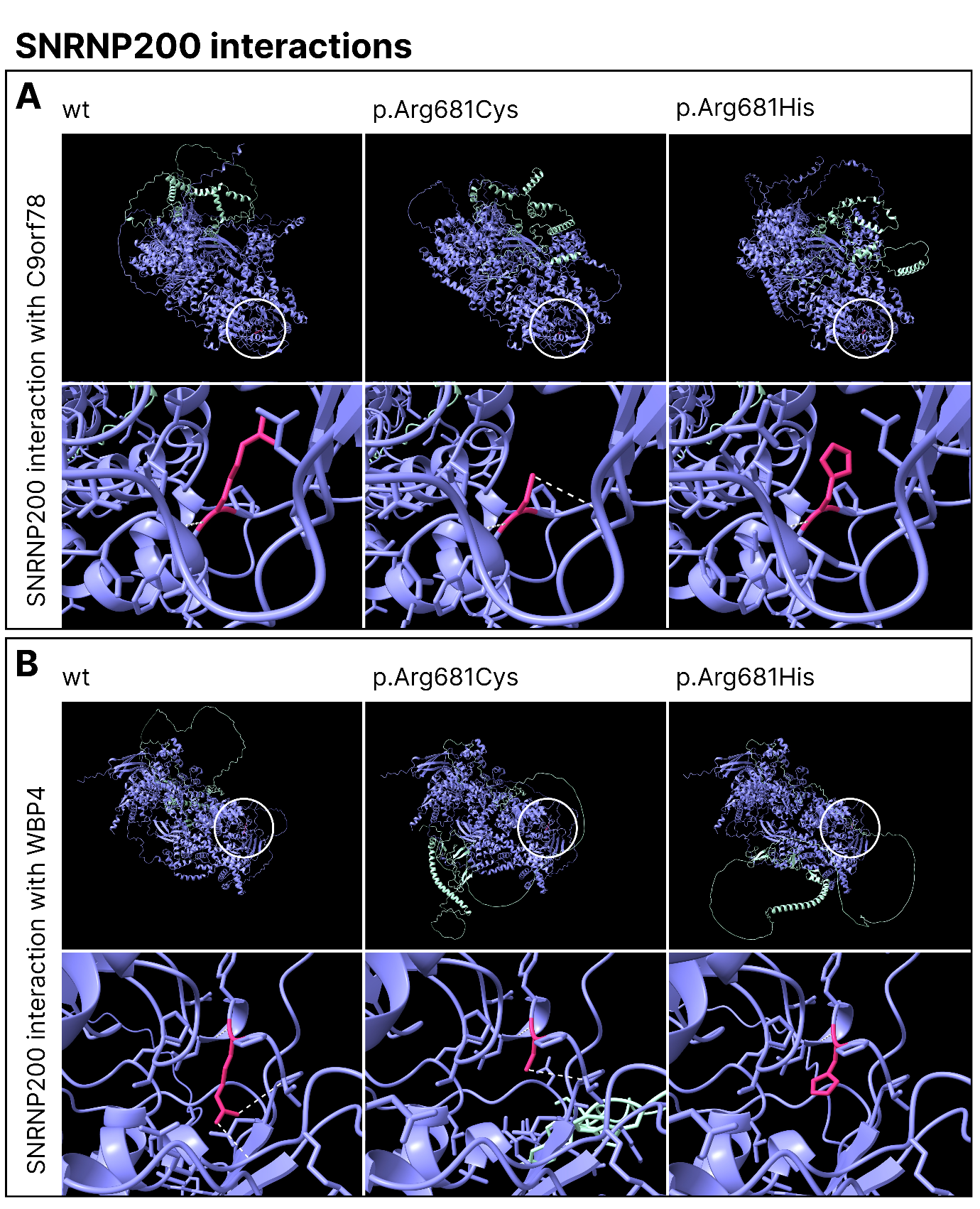
Figure 4. SNRNP200 AlphaFold multimer modelling**. A interaction of wildtype (WT) and variant (p.Arg681Cys and p.Arg681His) SNRNP200 with C9orf78 protein partner. B interaction of wildtype (WT) and variant (p.Arg681Cys and p.Arg681His) SNRNP200 with WBP4 protein. The residue of interest is coloured in pink. Hydrogen bonds are denoted by the white dashed lines and clashes are denoted by the purple dashed lines.


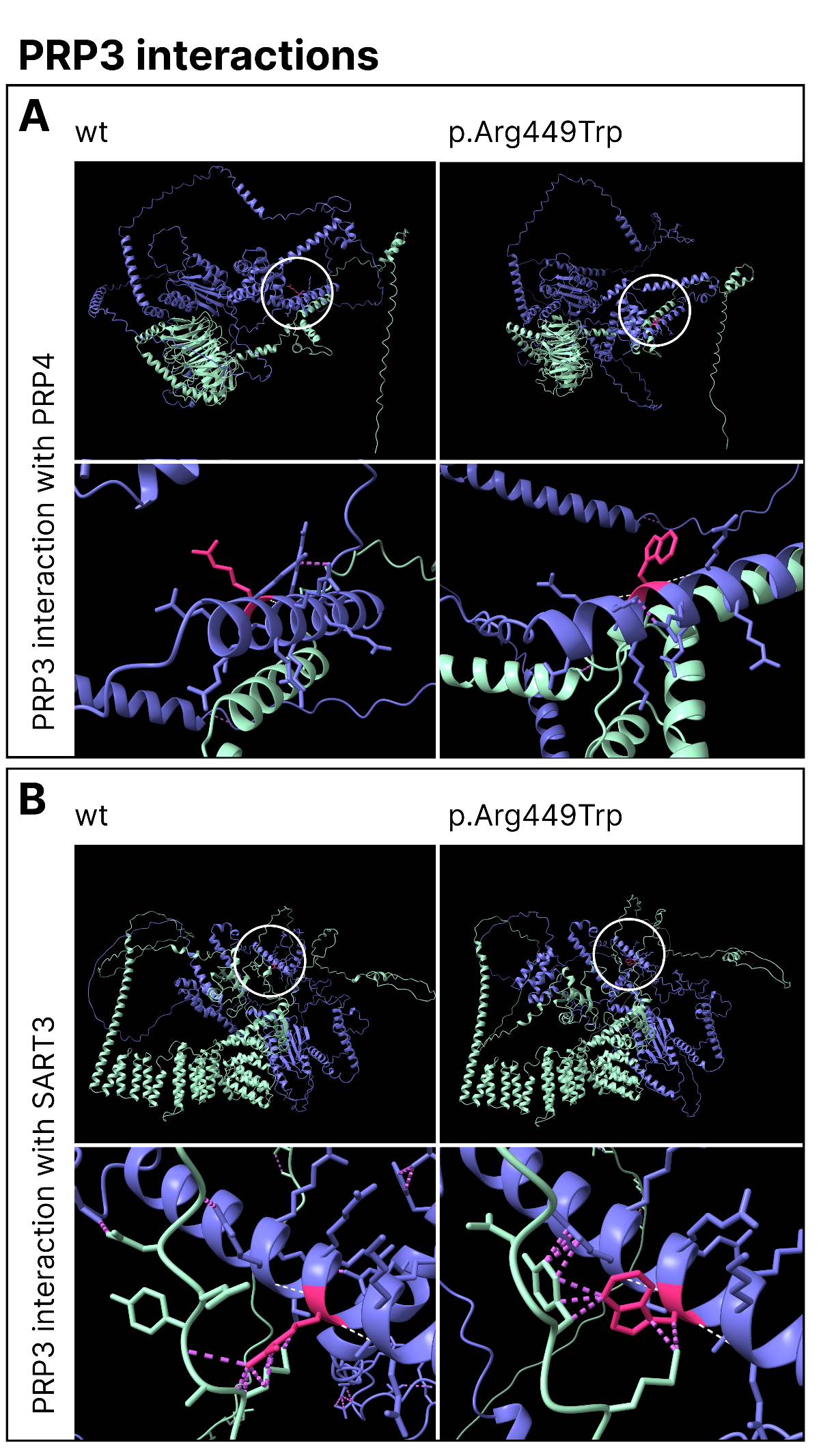


**Figure 5. PRP3 AlphaFold multimer modelling.** A interaction of wildtype (WT) and variant (p.Arg449Trp) PRP3 with PRP4 protein partner. B interaction of wildtype (WT) and variant (p.Arg449Trp) PRP3 with SART3 protein. The residue of interest is coloured in pink. Hydrogen bonds are denoted by the white dashed lines and clashes are denoted by the purple dashed lines.


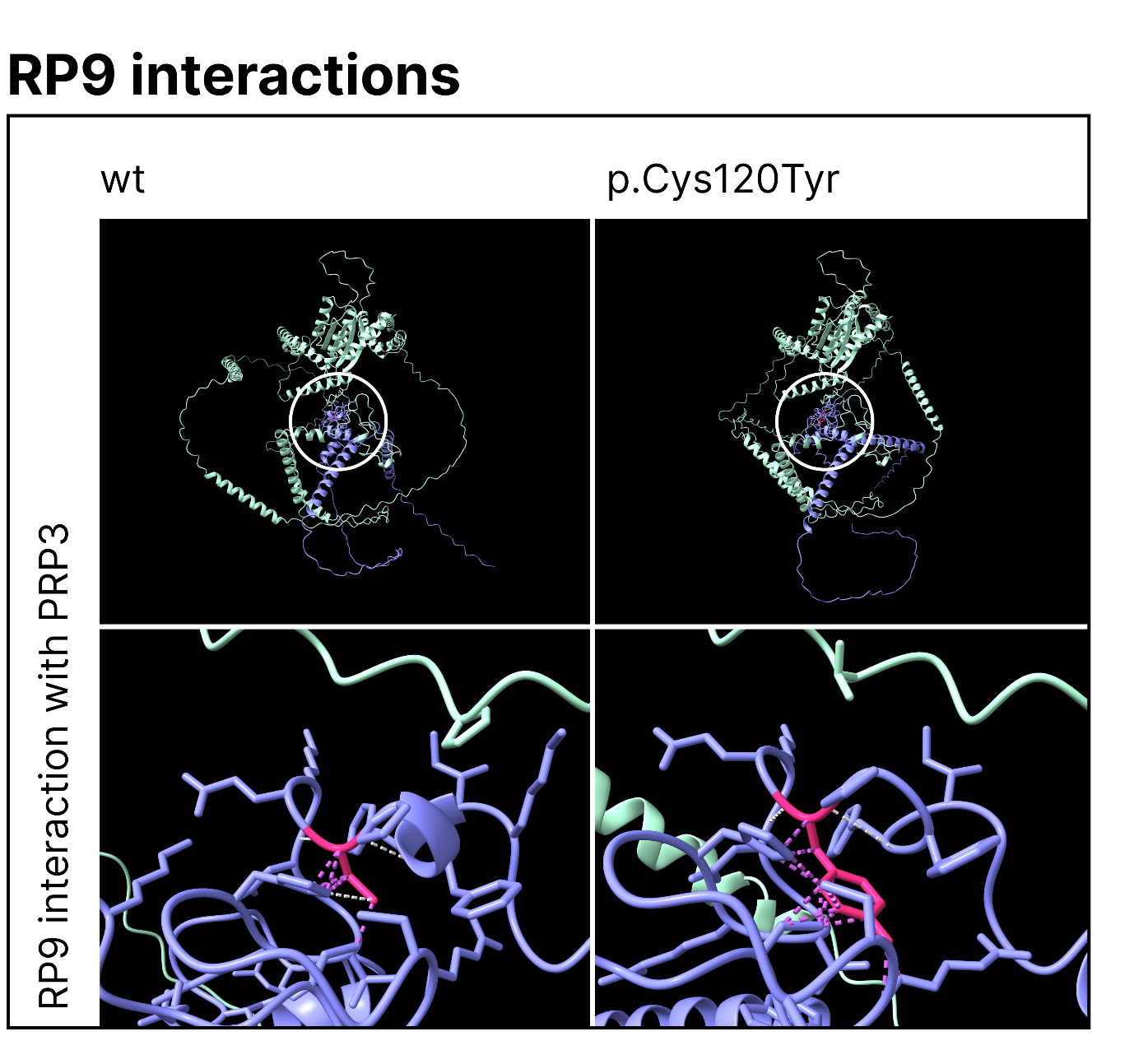


**Figure 6. RP9 AlphaFold multimer modelling.** Interaction of wildtype (WT) and variant (p.Cys120Tyr) RP9 with PRP3 protein partner. The residue of interest is coloured in pink. Hydrogen bonds are denoted by the white dashed lines and clashes are denoted by the purple dashed lines.

**
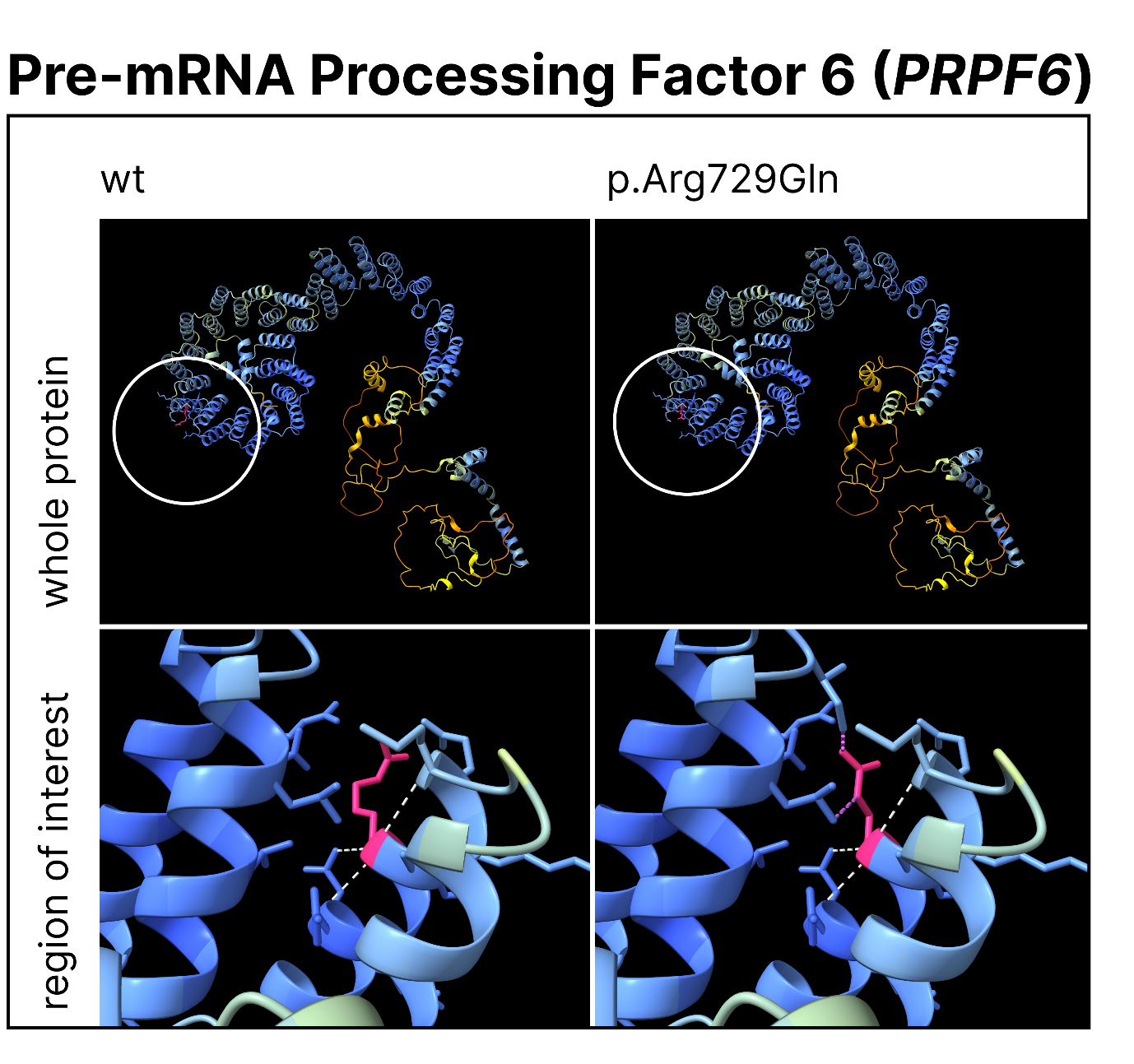
Figure 7. PRPF6 AlphaFold modelling.** Wildtype (WT) and variant (p.Arg729Gln) are coloured in pink representing the residue of interest. Hydrogen bonds are denoted by the white dashed line and clashes are denoted by the purple dashed line.
